# The application of artificial airway care for postoperative weaning patients with cerebral hemorrhage by failure mode and effect analysis mode

**DOI:** 10.3389/fmed.2025.1645973

**Published:** 2025-11-05

**Authors:** Xifei Tang, Jianhe Yue, Yao Chen, Runmei Zhou, Chanjuan Wang, Ting Zhang, Xiuni Gan

**Affiliations:** ^1^Department of Neurosurgery, The Second Affiliated Hospital of Chongqing Medical University, Chongqing, China; ^2^Department of Nursing, The Second Affiliated Hospital of Chongqing Medical University, Chongqing, China

**Keywords:** FMEA mode, artificial airway care, postoperative weaning patients, cerebral hemorrhage, application

## Abstract

**Objective:**

To investigate the application of artificial airway care under the Failure Mode and Effect Analysis (FMEA) mode in postoperative weaning patients with intracerebral hemorrhage (ICH).

**Methods:**

A total of 137 patients who met the inclusion and exclusion criteria were selected from an affiliated hospital of Chongqing Medical University. Using a random number table, 67 patients and 70 patients were randomly assigned to the control group and the experimental group, respectively. The control group received conventional care, while the experimental group received artificial airway care based on the FMEA mode for postoperative weaning patients with cerebral hemorrhage. Data was analyzed using SPSS 27.0 to compare the incidence of pulmonary infection, re-intubation, aspiration, sputum viscosity, and tracheal tube duration between the two groups.

**Results:**

The incidence of pulmonary infection, re-intubation, and aspiration in the experimental group was significantly lower than that of the control group (*p* < 0.05). After intervention, the state of sputum viscosity in the experimental group was significantly improved compared to the control group (*p* < 0.05). The PaO2 level in the arterial blood gas analysis was significantly higher in the experimental group after intervention (*p* < 0.05). Additionally, the tracheal extubation time in the experimental group was significantly shorter than that of the control group (*p* < 0.05).

**Conclusion:**

Artificial airway care under the FMEA mode can effectively reduce the complications in postoperative weaning patients with cerebral hemorrhage, such as pulmonary infections, re-intubation, and aspiration. It can improve PaO_2_ levels and sputum viscosity in arterial blood gas analysis while reduce the tracheal tube duration, and ultimately promote patient recovery.

## 1 Introduction

Intracerebral hemorrhage (ICH), non-traumatic in origin, refers to spontaneous bleeding within the brain parenchyma. Most cases are caused by the rupture of blood vessels due to hypertensive small artery sclerosis. Hence, it is referred to as hypertensive intracerebral hemorrhage. It is one of the leading causes of death and disability among the population (1). Patients with ICH represent a particularly challenging cohort due to the abrupt and often massive nature of the hemorrhage, which leads to significant primary and secondary brain damage. These patients frequently present with severe and dynamic neurological impairments, complicating their postoperative care, especially during critical periods such as weaning from mechanical ventilation. Currently, clinical treatments for ICH mainly include conservative management and surgical evacuation of the hematoma (2). Patients requiring surgical hematoma evacuation usually have severe disease and heavy bleeding. Postoperatively, patients may not be able to remove the artificial airway in time after weaning due to impaired consciousness or lung infection nadequate airway management during the period between weaning and extubation usually results in the need for reintubation and mechanical ventilation, leading to prolonged hospitalization and increased financial burden. Therefore, the quality of airway care after weaning in postoperative ICH patients has a direct impact on the successful removal of the tracheal tube.

Previous studies (3–6) on airway management for patients with artificial airways during the period between weaning and decannulation primarily focus on single factors, such as airway humidification, oxygen therapy methods, and pulmonary rehabilitation guidance. However, there is a lack of systematic and refined nursing care specific to the weaning and pre-decannulation phases. For example, there is a lack of systematic guidance on how to select appropriate humidification and oxygen therapy methods for different patients, how to implement pulmonary rehabilitation and evaluate its effectiveness after weaning, and how to enforce aspiration prevention measures, airway suctioning protocols, and cuff pressure monitoring procedures. Therefore, a systematic nursing process is needed to guide nurses in providing effective care in clinical practice. In the context of postoperative airway management in weaned ICH patients, multiple deficiencies may exist at different stages of care. These deficiencies need to be identified and analyzed using specific methods to pinpoint the causes and facilitate targeted improvements and refinements.

Failure Mode and Effect Analysis (FMEA) is a proactive risk assessment and management method that systematically evaluates potential failures in a process, quantifies the risks, identifies the potential causes of failures, and formulates corrective actions to establish standardized procedures. This approach is effective in preventing or reducing the occurrence of problems (7). FMEA has been widely used to optimize medical processes in hospitals due to high applicability in improving healthcare quality and reducing errors (8). Although FMEA has been widely adopted in healthcare for process improvement, its targeted application in the high-risk, specific, and often overlooked clinical window between weaning and extubation in Postoperative Patients with Cerebral Hemorrhage remains novel. Previous FMEA studies on nursing focus on broader clinical processes. In contrast, this study delves into the granular, operational-level deficiencies in airway management during this critical period.

In this study, FMEA risk management approach was used to proactively evaluate and quantify the failure points in airway management for postoperative ICH patients following weaning. The causes of these failures were analyzed to develop corrective measures. Evidence-based practices were then employed to refine nursing processes, with quality control management teams ensuring the effective implementation of these processes. This study aimed to improve the quality of clinical nursing care, ensure patient safety, and promote early recovery in patients.

## 2 Materials and methods

### 2.1 Clinical data

A sample size analysis was conducted before the study. The sample size was calculated using the PASS 15.0 software, and the sample size calculation was chosen to compare two sample rates. At a significance level of *α* = 0.05 and *β* = 0.10, literature review and reference to similar studies indicate that the incidence of pulmonary infection in conventional airway management is 36.07%, while the incidence in bundle airway management is 11.48%. The calculation indicated N = 58. Considering a 10–15% dropout rate, the sample size for both groups should be set at least 128 cases, comprising 64 cases in the experimental group and 64 cases in the control group.

Patients who underwent postoperative weaning following intracerebral hemorrhage (ICH) surgery at an affiliated hospital of Chongqing Medical University between October 1, 2022, and September 30, 2024, were selected for this study. We used the random assignment for grouping. Using a computer, we generated 140 random decimal numbers in Excel. Here, the vales greater than 0.5 was assigned to the experimental group, while the values less than 0.5 was assigned to the control group. Under the hidden distribution rules, the researchers allocated the enrolled subjects to the corresponding groups based on their serial numbers. Finally, 67 patients were selected as the control group, with the remaining 70 patients assigned to the experimental group.

*Inclusion criteria included* age ≥ 50 years; diagnosis of intracerebral hemorrhage confirmed by imaging and treated surgically according to the Chinese Guidelines for the Diagnosis and Treatment of Intracerebral Hemorrhage; post-operative patients with an artificial airway after weaning; stable vital signs post-surgery; and family members signed informed consent.

*Exclusion criteria* involved patients with chronic obstructive pulmonary disease or autoimmune diseases; patients with Intracranial Infection; **i**ncomplete clinical data; end-stage disease patients; and patients discharged with an artificial airway or deceased. This study was approved by the hospital’s Medical Ethics Committee (Grant No. 2024-EC-73).

### 2.2 Methods

#### 2.2.1 Control group

The control group received conventional artificial airway care interventions after weaning: (1) use of Venturi + heated humidifier oxygen therapy, with close monitoring of airway sputum viscosity; (2) suctioning performed as needed according to central negative pressure suction standards, with cuff pressure monitored every 4 h and maintained between 25 and 30 cmH_2_O; (3) routine oral care 3–4 times per day; and (4) routine caregiver education on turning and back-patting methods to assist with effective sputum clearance.

#### 2.2.2 Experimental group

On top of traditional airway care interventions, the experimental group received an airway care process based on the FMEA mode for patients after weaning. A quality control management team supervised the clinical implementation of the process. This study was informed by relevant domestic and international literature (9–12). The main steps were as follows:

*Team formation*: A research team of 10 members was formed with clear responsibilities including doctors, nurses, and pulmonary rehabilitation therapists. All team members received FMEA method training.
*Flowchart creation*: This team used brainstorming sessions to illustrate the airway care process for postoperative ICH patients in flowchart format. The process included six key steps: catheter fixation, selection of humidified oxygen therapy, airway suction, pulmonary rehabilitation, cuff pressure monitoring, and extubation preparation.
*Failure mode and effect analysis*: The team identified failure modes within the process and the potential causes of these failures. The failure modes for each of the six steps were numbered, and a table was created for failure cause and impact analysis. Each failure mode was scored for Risk Priority Number (RPN), calculated by the formula: RPN = Severity (S) × Occurrence (O) × Detection (D). A higher RPN value indicates a greater impact. The RPN values range from 7.3 to 12; 8 items were greater than 9, amounting to 57% of the total. The detailed scoring criteria are shown in Tables 1–3. We obtained a K value of 0.68 using Fleiss’ Kappa, indicating a good level of inter-rater reliability. This study recommends actions to reduce failure likelihood in artificial airway care for postoperative weaning patients with cerebral hemorrhage when the RPN exceeds 9.
*Process refinement*: Based on the FMEA analysis, the team conducted a literature review of domestic and international practices for airway care after weaning, including aspects like catheter fixation, oxygen therapy, airway suction, pulmonary rehabilitation, cuff pressure monitoring, and extubation preparation. The findings were integrated with departmental conditions to develop an actionable airway care process.

**Table 1:** Severity (S) scoring criteria.

Severity level	Score	Scoring criteria
Extreme	4	Extubation failure, requiring reconnection to ventilator
Major/High	3	Extubation very likely to fail, highly likely to require reconnection to ventilator
Moderate	2	Extubation may fail, can be avoided with slight attention to prevent reconnection to ventilator
Minor/Low	1	Low possibility of extubation failure,still needs to be prevented

**Table 2:** Occurrence (O) scoring criteria.

Occurrence level	Score	Scoring criteria
Very High/Frequent	4	Could happen every shift
High/Occasional	3	Could happen daily
Moderate/Uncommon	2	Could happen weekly
Low/Remote	1	Rarely happens

**Table 3:** Detection (D) scoring criteria.

Detection level	Score	Scoring Criteria
Almost impossible	4	No process controls, cannot detect
Low	3	Some process controls, detection < 50%
Moderate	2	Process controls in place, detection 51–89%
High	1	Process controls in place, detection 90–100%

#### 2.2.3 FMEA-based nursing process and expert consultation

According to the literature review and screening (5, 13–26), a preliminary airway care process for postoperative ICH patients was summarized. Face-to-face discussions were carried out to reach a consensus on the process. The airway care implementation process was divided into six dimensions with 18 key items.

Catheter fixation and maintenance (3 items), selection of oxygen therapy and humidification modes (3 items), airway suction (2 items), prevention of aspiration (3 items), pulmonary rehabilitation (2 items), extubation evaluation and implementation (5 items), a questionnaire for expert consultation was created based on the team’s initial management process list. The questionnaire included expert demographics, instructions, influencing factors, and importance scoring. Using a Likert 5-point scale, experts rated the importance of each item (5 = very important, 1 = very unimportant). The experts were selected based on the following criteria: work experience ≥ 10 years; professional title: head nurse or higher, preferably at the associate senior level or above; educational background: Bachelor’s degree or higher, preferably Master’s or above; and expertise: Neurosurgery, ICU, evidence-based nursing, etc.

A total of 22 experts from 12 top-tier hospitals in Chongqing, Sichuan, and Guizhou participated in this study, including 15 associate senior or higher-level experts, 7 intermediate-level experts, 18 nursing managers, 2 respiratory therapists, and 2 senior nursing staff. After two rounds of expert consultation, two additional items were added: Assessment of sedation and analgesia (2 items), and details were adjusted according to expert feedback. The final process included 20 items. The airway care process based on the FMEA mode is summarized in Table 4.

**Table 4:** Post-operative artificial airway nursing measures for weaning patients with cerebral hemorrhage.

Main process	Sub-process	Process details
Catheter fixation and maintenance	Confirm fixation	Ensure endotracheal, nasotracheal, or tracheostomy tubes are securely fixed using professional fixation devices or self-cut tape and cotton bands for dual secure fixation.
	Dynamic assessment	Check the fixation every hour to ensure the catheter is not loose, and adjust as needed.
	Dressing changes	Perform sterile dressing changes at the tracheostomy site twice daily.
Oxygen therapy and humidification	Oxygen therapy and humidification selection	After disconnection, the active heating and humidification system should be selected as the first choice. High-flow humidified oxygen therapy (HFNC) is the preferred option. If equipment resources are limited, a servo-type heating humidifier combined with a Venturi device can be used for oxygen therapy humidification. The temperature should be set at 31–37 °C, and the absolute humidity should be controlled to be higher than 30 mg H₂O/L. (38–43)
	Sputum assessment	Assess sputum consistency each shift: Grade I - thin, white, like rice soup, mildly viscous; Grade II - moderately viscous, retains small amount of sputum after suctioning; Grade III - thick, yellow, purulent, large amount of sputum retained after suctioning.
Airway suctioning	Suction indications	Suction when visible sputum is present, wheezing is heard, oxygen saturation drops below 95%, or if rales are heard in both lungs. Also, suction when aspiration is suspected or if the patient cannot effectively cough up sputum. If none of these situations occur, suction once per shift for assessment and to stimulate cough reflex.
	Procedure compliance	Follow airway suctioning procedure strictly.
Prevention of aspiration	Identify high-risk patients	High-risk patients include the elderly, those with GCS < 9, head of bed < 30°, feeding rate ≥ 40 mL/h, gastric residual volume ≥ 150 mL, vomiting status, use of sedatives, muscle relaxants, or anticholinergic drugs, and those with gastrointestinal diseases.
	Enteral nutrition care	Includes assessing enteral nutrition tolerance in high-risk patients, positioning management, post-pyloric feeding, and preventing and managing gastric retention.
	Cuff pressure monitoring	Correctly monitor cuff pressure every 4 h. When suctioning or changing position, recheck and adjust cuff pressure, and record findings.
Restraint and sedation/pain assessment	Restraint evaluation	Evaluate using a restraint scale. Restraints are not applied if muscle strength ≤ 2 or the patient is in moderate or deep coma. If score ≥ 29, restraints are applied, reassessed every 2 h, and released for 15 min each time. Assess if restraints can be removed every 8 h or if the patient’s condition changes.
	Sedation/pain evaluation	Perform sedation and pain assessments according to patient condition and physician orders. Use shallow sedation for easier monitoring of consciousness, muscle strength, and weaning condition.
Pulmonary rehabilitation	Pulmonary physiotherapy	Develop individualized, goal-oriented pulmonary physiotherapy plans (44), including respiratory endurance training, airway clearance techniques, and diaphragm stimulation for awake patients, twice a day.
	Assisted sputum clearance	Perform nebulization 3–4 times a day, assist with turning and chest percussion every 2 h, guide in executing these techniques, and perform mechanical sputum clearance twice a day as needed
Extubation evaluation and procedure	Assess the need for tube retention	Assess daily whether extubation is necessary based on stable vital signs, strong cough, and clear airway.
	Withdrawal criteria	Withdrawal Criteria: stable vital signs, strong cough, PaO2 ≥ 60 mmHg without oxygen, PaCO_2_ < 50 mmHg, no airway obstruction, no recent surgery, and patient consent. Evaluate for HAP/VAP risk and if oxygenation is normal (PaO2 ≥ 60 mmHg or PaO2/FiO2 ≥ 300).
	Psychological care and education	Educate the patient and family on the significance of early extubation, the procedure, and the cooperation required to alleviate anxiety.
	Extubation procedure	Perform a leak test, deflate the cuff, and gradually remove the tube while suctioning the airway. If the leak test is unsuccessful, use medication (e.g., methylprednisolone) and extubate after 10 min.
	Post-extubation monitoring	Monitor for complications such as sore throat, swallowing difficulty, or oxygen saturation drops. If oxygen saturation decreases, administer high-flow oxygen therapy (HFNC) and be prepared for reintubation.

#### 2.2.4 Establishing a quality control management system

Firstly, a quality control management team was established, consisting of research members from the project team. The team would implement a three-level nursing management system: head nurse - nursing group leader - responsible nurse. The team consists of 7 members, including 2 head nurses, 4 nursing group leaders, and 1 assistant head nurse. Team members would analyze the issues in airway management of patients after weaning. Define clear nursing objectives and ensure effective implementation. The nursing group leader would complete the “Postoperative Airway Management Daily Inspection Form for Hemorrhagic Stroke Patients After Weaning,” ensuring the effective implementation of nursing procedures during weaning, thus improving the quality of patient care. Evaluate the results of the objectives. During the implementation phase, regular team meetings were performed to continuously evaluate and adjust the plan to achieve the expected objectives.

### 2.3 Observational indicators

The airway sputum viscosity between the two groups of patients was compared. The criteria for assessment are shown in Table 4.

The blood gas analysis indicators between the two groups of patients were compared: Blood gas analysis would be performed on PaO_2_ and PaCO_2_ values once within 24 h after weaning and once before extubation.

The incidence of pulmonary infections between the two groups of patients was compared. Diagnostic criteria for pulmonary infection: Chest X-ray shows pulmonary infiltration shadows or new inflammatory lesions; signs of pulmonary consolidation and/or the presence of wet sounds during lung auscultation, with one of the following conditions: (1) Peripheral blood white blood cell count >10×10^9/L or <4×10^9/L, with or without nuclear shift; (2) Fever, body temperature >37.5 °C, and large amounts of purulent respiratory secretions; and (3) New pathogens isolated from bronchial secretions. Ventilator-Associated Pneumonia (VAP) needs to be excluded, which refers to pneumonia that occurs 48 h after mechanical ventilation or within 48 h after extubation. This determination was performed through a combined assessment of the temporal window and clinical context.

The reintubation rates between the two groups were compared, specifically the reintubation rate within 24 h after weaning.

The duration of the endotracheal tube in place between the two groups was compared, specifically the time from successful weaning to the removal of the endotracheal tube.

### 2.4 Statistical methods

Statistical analysis was conducted using SPSS 27.0 software. Count data is expressed as frequency and percentage. Group comparisons were performed using the chi-square test. Measurement data is expressed as mean ± standard deviation (c ± s). The corresponding group comparisons were conducted using the t-test. A *p*-value of <0.05 is considered statistically significant.

## 3 Results

### 3.1 Comparison of general information

We used the random assignment for grouping. A total of 137 patients were included. Sixty-seven patients (46 males and 21 females with an average age of 58.40 ± 12.14 years) were randomly selected as the control group. Seventy patients were assigned to the experimental group, consisting of 44 male and 26 female patients with an average age of 56.34 ± 11.99 years. There were no statistically significant differences between the two groups in terms of age, sex, hemorrhagic stroke location or bleeding volume (*p* > 0.05), as shown in Table 5.

**Table 5:** General information of patients.

Item	Control group (*n* = 67)	Experimental group (*n* = 70)	Statistic	*p*
Sex			*X*^2^ = 1.117	0.291
Male	46	42		
Female	21	28		
Age	58.40 ± 12.14	56.34 ± 12.00	*t* = 0.999	0.320
bleeding volume	28.99 ± 10.12	26.81 ± 8.93	*t* = 1.333	0.185
Bleeding site			FET = 1.277	0.960
Basal ganglia	30	29		
Brainstem	2	3		
Ventriculus cerebri	9	12		
Lobe	17	19		
Thalamus	3	3		
Cerebellum	6	4		

X^2^ = chi-square test; *t* = *t*-test; FET, Fishers’ exact test.

### 3.2 Comparison of complication incidence between the two groups

The experimental group adopted the artificial airway process based on the FMEA model. The rates of pulmonary infection, re-mechanical ventilation, and aspiration were all lower than those in the control group (*p* < 0.05), and the differences were statistically significant (Table 6).

**Table 6:** Comparison of complication rates.

Group	Sample size	Pulmonary infection rate	Secondary mechanical ventilation rate	Aspiration rate
Control group	67	12 (17.91%)	8 (11.94%)	7 (10.44%)
Experimental group	70	2 (2.85%)	2 (2.85%)	1 (1.43%)
*χ* ^2^		8.455	4.174	5.065
*p*		0.003	0.041	0.027

### 3.3 Comparison of sputum viscosity before and after intervention in both groups

Before the intervention, there was no significant difference in sputum viscosity between the two groups. However, a significant difference was observed after the intervention (*p* < 0.001). In the control group, sputum viscosity showed slight improvement post-intervention, but the change was not as pronounced as in the experimental group. In contrast, the experimental group demonstrated a highly significant improvement in sputum viscosity after the intervention (*p* < 0.001), with a marked increase in the number of patients reporting no sputum or grade I sputum, and a complete absence of grade III sputum. These results indicate that the intervention in the experimental group was effective in reducing sputum viscosity compared to the control group (Table 7).

**Table 7:** Comparison of phlegm viscosity.

Group	Sputum characteristics before intervention				Sputum characteristics after intervention				*H*	*p*
	Absence	I degree	II degree	III degree	Absence	I degree	II degree	III degree		
Control group (*n* = 67)	0	9	38	20	2	18	39	8	9.460	0.02
Experimental group (*n* = 70)	2	14	38	16	15	33	22	0	38.928	<0.001
*h*	2.153				26.050					
*p*	0.142				<0.001					

H, Kruskal-Wallis H; *p*, Probability value.

### 3.4 Comparison of PaO_2_ and PaCO_2_ before and after intervention between the two groups

Before the intervention, there was no statistically significant difference in PaO_2_ and PaCO_2_ between the two groups (*p* > 0.05). After the intervention, PaO_2_ decreased in both groups. This might be related to the changes in the oxygen therapy mode and the decrease in oxygen flow during the weaning process. However, the PaO_2_ values in the experimental group were significantly higher than those in the control group, with a statistically significant difference (*p* < 0.05). The results showed that the artificial airway care for postoperative weaning patients with cerebral hemorrhage by FMEA could improve the PaO_2_ of patients. There was no statistically significant difference in PaCO_2_ between the two groups before and after the intervention (*p* > 0.05; Table 8).

**Table 8:** Comparison of blood gas index PaO_2_ AND PACO_2_.

Item		Control group (*n* = 67)	Experimental group (*n* = 70)	*t*	*p*
PACO_2_	Pre-intervention	37.64 ± 4.19	37.09 ± 3.92	1.054	0.294※
	Post-intervention	37.19 ± 4.32	35.84 ± 4.17	1.797	0.075※
PAO_2_	Pre-intervention	142.92 ± 54.34	130.17 ± 44.24	–	0.348‡
	Post-intervention	95.24 ± 19.22	104.43 ± 19.78	2.701	0.008※

※ Unpaired *t* test; ‡ Mann–Whitney test.

### 3.5 Comparison of endotracheal tube duration between the two groups

The comparison of endotracheal tube duration between the two groups revealed a statistically significant difference. The experimental group had a significantly shorter duration of tracheal catheter placement after weaning compared to the control group (p<0.001), indicating a highly significant reduction in tube duration in the experimental group, as shown in Table 9.

**Table 9:** Comparison of the time to carry tracheal catheter.

Group	Sample size	Time to carry the tracheal catheter
Control group	67	7.73 ± 10.13
Experimental group	70	3.91 ± 5.02
*t*		2.817
*p*		<0.001

## 4 Discussion

### 4.1 The challenge of airway management after weaning

Patients after hemorrhagic stroke often experience prolonged endotracheal intubation due to some factors such as suppression of the respiratory center, neurogenic brain edema, or consciousness impairment. Although weaning from the ventilator may be attempted once weaning criteria are met, artificial airway management following ventilator weaning remains a critical component prior to extubation. Existing studies (5, 27–32) indicate a lack of high-quality evidence regarding airway management strategies during this phase, leading to inconsistent clinical practices. Inadequate airway care during this period can lead to extubation failure, reintubation, and prolonged intubation time.

### 4.2 Application of FMEA in airway care

This study innovatively introduced FMEA, a proactive risk management tool, to systematically optimize the artificial airway care process for postoperative weaning patients with cerebral hemorrhage. Using FMEA, we identified and quantified potential failure modes in the nursing process, implemented corrective measures, and established standardized protocols. The results demonstrated that the FMEA-based nursing protocol significantly reduced the incidence of pulmonary infection, reintubation, and aspiration. This improvement can be attributed to the refined management of key aspects such as airway humidification, oxygen therapy mode selection, aspiration prevention, and pulmonary rehabilitation, ensuring consistency and quality in care delivery. Studies by Li et al. (33) and Qu et al. (34) also support the efficacy of FMEA in reducing ICU-acquired infections and multidrug-resistant organism infections, aligning with our findings and underscoring the broad applicability of FMEA in nursing quality management.

### 4.3 Broader implications and applicability of FMEA

This study focused on a high-risk yet often overlooked population—postoperative weaning patients with cerebral hemorrhage—providing both empirical evidence and a practical workflow for airway management. As a systematic and proactive methodology, FMEA has demonstrated value across various nursing contexts. Studies by Chen et al. (35) and Wang et al. (36) reported its successful application in preventing intraoperatively acquired pressure injuries and enteral nutrition-related diarrhea, Ding et al. (7) and Liu et al. (8) respectively reported their applications in bedside handover of postoperative pipeline care and proactive healthcare risk evaluation, consistent with our approach. It is worth emphasizing that the effective implementation of FMEA requires sustained quality control and multidisciplinary collaboration to ensure adherence and continuous improvement. Furthermore, our findings help address the unmet needs in neurotrauma care identified by Dasic et al. in their recently proposed management matrix for neurotrauma centers (37). By employing a proactive risk management strategy (FMEA) to optimize airway management during the critical weaning-to-extubation phase, this study introduces a structured and systematic nursing framework. This approach aligns with the call for more standardized and refined clinical processes in specialized neurotrauma care. Therefore, our work not only reinforces the evidence base for postoperative intracerebral hemorrhage (ICH) management but also offers a practical methodology to improve nursing quality and patient safety, in keeping with the current priorities of neurotrauma centers.

### 4.4 Study limitations

This study has several limitations. First, the data were derived from a single center in Chongqing, which may limit generalizability. Future multi-center studies with larger samples are needed to validate the effectiveness and scalability of the proposed protocol. Second, although this study focuses on key failure modes, further optimization of ancillary processes can enhance overall airway care quality.

## 5 Conclusion

In conclusion, by introducing the FMEA model, this study developed a scientific, systematic, and operable artificial airway care protocol for postoperative weaning patients with cerebral hemorrhage. Implemented through a three-tier quality control system, this protocol significantly improved nursing quality, reduced complication rates, enhanced blood gas parameters and sputum viscosity, shortened endotracheal intubation duration, and ultimately promoted patient recovery.

## Data Availability

The raw data supporting the conclusions of this article will be made available by the authors, without undue reservation.
